# Nucleic acid strategies for infectious disease treatments: The nanoparticle-based oral delivery route

**DOI:** 10.3389/fphar.2022.984981

**Published:** 2022-08-29

**Authors:** Fengqian Chen, Qi Liu, Yang Xiong, Li Xu

**Affiliations:** ^1^ Translational Research Program, Department of Anesthesiology and Center for Shock Trauma Anesthesiology Research, University of Maryland School of Medicine, Baltimore, MD, United States; ^2^ Department of Dermatology, Johns Hopkins University School of Medicine, Baltimore, MD, United States; ^3^ College of Pharmaceutical Science, Zhejiang Chinese Medical University, Hangzhou, China; ^4^ Department of Anorectal Surgery, the First Affiliated Hospital of Zhejiang Chinese Medical University, Hangzhou, China

**Keywords:** nucleic acid, nanoparticles, targeted delivery, IBD, COVID-19

## Abstract

Therapies based on orally administrated nucleic acids have significant potential for the treatment of infectious diseases, including chronic inflammatory diseases such as inflammatory bowel disease (IBD)-associated with the gastrointestinal (GI) tract, and infectious and acute contagious diseases like coronavirus disease 2019 (COVID-19). This is because nucleic acids could precisely regulate susceptibility genes in regulating the pro- and anti-inflammatory cytokines expression related to the infections. Unfortunately, gene delivery remains a major hurdle due to multiple intracellular and extracellular barriers. This review thoroughly discusses the challenges of nanoparticle-based nucleic acid gene deliveries and strategies for overcoming delivery barriers to the inflammatory sites. Oral nucleic acid delivery case studies were also present as vital examples of applications in infectious diseases such as IBD and COVID-19.

## 1 Introduction

Human genome diseases have been highly discussed and researched into in recent decades. Disease-related genetic changes are being studied, inspiring various discoveries on gene therapeutics. Viral and non-viral formulations are developed to deliver therapeutics effectively and safely for gene editing. Compared to viral deliveries, the non-viral system demonstrated safety and efficiencies in extensive cargo loading. Plasmid deoxyribonucleic acid (DNA)-based antigen vaccines or therapeutic proteins are broadly developed (Rothman et al., 2005; Nurunnabi et al., 2017; Sinha et al., 2017), as well as ribonucleic acid (RNA)-based inhibitors/stimulators such as antisense oligonucleotides (ASOs), short interfering RNA (siRNA) (Jain et al., 2017; Vaidya et al., 2019), microRNA (miRNA) (Brown et al., 2018), messenger RNA (mRNA) (Sago et al., 2018) or aptamer (Chen et al., 2017a). These nucleic acids function in modifying or regulating the genome and consequently the repair of genetic defects. Moreover, they can also be applied to the silencing of specific gene expressions.

The oral delivery route is broadly applied to administer and deliver therapeutics onsite. The leading target site of this delivery method is the gastrointestinal (GI) tract. GI tract represents a large surface area, facilitating nucleic acids’ uptake with its associated massive microvessels to deliver cargo by the blood vessels into other tissues. The most desirable targets for oral delivery systems would be gut chronic inflammatory diseases, such as ulcerative colitis (UC), Crohn’s disease, and GI infections. Another potential application of oral administration drugs poses the delivery of plasmid DNA (pDNA) or mRNA-based vaccines to the GI tract, where the GI tract greatly facilitates cargo absorption and delivery to the whole body through mucosal.

To our knowledge, oral drug formulations are the most common drug administration method. It greatly benefits therapeutics’ non-invasive, convenient, and safe administration, lowers manufacturing costs, and improves patients’ appliance (Fattal and Fay, 2021). Moreover, orally administrated gene therapy would go a long way for chronic inflammations, especially for inflammatory bowel disease (IBD) and acute infections like coronavirus disease 2019 (COVID-19). However, over the last decade, the progress made toward the clinical translation of delivering nucleic acid-based oral therapeutics has been limited due to various barriers to the GI tract (O’Driscoll et al., 2019). Herein, these challenges and opportunities are further enclosed.

## 2 Nucleic acid as the cargo

Nucleic acid-based gene therapy is based on therapeutic molecules DNA or RNA and aims to achieve multiple goals *in vivo*. Firstly, to deliver siRNA or miRNA for gene downregulations. Secondly, to deliver DNA or mRNA for gene overexpression. Thirdly, to activate specific immune responses by delivering pattern-recognition receptor (PRR)s-stimulating nucleic acids (Barbalat et al., 2011; Wang et al., 2020). Herein, we briefly compared these therapeutic strategies according to their common characteristics (Table 1).

**TABLE 1:** The characteristics’ comparison of nucleic acid-based therapeutics.

Characteristics	pDNA	siRNA	miRNA	mRNA	ASO
mechanism of action	specific gene expression; specific gene replacement	specific gene knockdown	specific gene regulation	specific gene expression	specific gene knockdown
action site	cell nucleus	cytoplasm	cytoplasm	cytoplasm	cytoplasm
chemical property	double-stranded circular DNA long molecules	double-stranded RNA (dsRNA) short molecules	small noncoding RNA short molecule	single-stranded long RNA molecule	short strand of deoxyribonucleotide analog
delivery challenges	large size; endosomal escape; entry into nucleus; off-target effect	endosomal escape; release into the cytoplasm; off-target effect	endosomal escape; release into the cytoplasm; nonspecific gene effect; off-target effect	endosomal escape; release into the cytoplasm	endosomal escape; release into the cytoplasm

**FIGURE 1:**
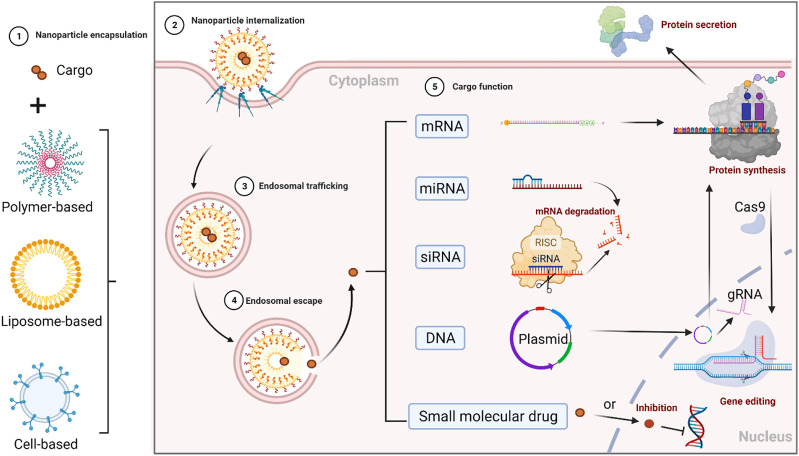
Delivery route and mechanism of gene regulations. 1) nucleic acids (cargo) are encapsulated in nanocarriers for extracellular trafficking, 2) after crossing the cell membrane, nanoparticles (NPs) are cellular internalized and uptake into the endosome, 3) NPs’ endosomal trafficking, 4) NPs’ endosomal escape into the cytoplasm, 5) nucleic acids’ separation from nanocarriers, and their functions at the target sites. Their functions vary but are not limited to: a) protein translation (mRNA in ribosome is decoded into the polypeptide or a protein); b) gene regulation (miRNA and siRNA could regulate specific gene expressions); c) gene transcription (pDNA in the nucleus is actively transcribed into mRNA and translated into the protein it encodes for *via* the same protein expression machinery the cell uses to produce the protein that it needs to function correctly. For instance, during the gene editing process in the CRISPR-Cas9 system, a short guide RNA (gRNA) sequence in this two-component complexed system guides the Cas9 endonuclease to the target site. The Cas9 endonuclease can be transcripted and translated from pDNA or be directly translated from mRNA. In addition, small molecular drugs such as transcription or translation inhibitors could also effectively inhibit gene expression).

A plasmid with a double-stranded circle (consisting of promoter and genes of interest) is often utilized in directing the overexpression of specific genes. However, there are several barriers that DNA faces to attaining cell-specific gene expression. Firstly, DNA must cross the plasma membrane and the nuclear envelope to deliver itself inside the nucleus (Liu et al., 2018a). Then in the cell nucleus, DNA containing the gene of interest needs to be transcribed into mRNA. Lastly, mRNA is exported into the cytoplasm and translated into the protein of interest (Lechardeur et al., 2005). The delivery route and mechanism of gene regulations are visualized in Figure 1.

On the other hand, single-stranded mRNA could simplify the transcription step in the nucleus and be directly delivered into the cytoplasm and translated into the protein of interest. Unlike pDNA, mRNA works in the cytoplasm with no need for entry to the nucleus, thus simplifying the delivery path of nucleic acids (Kauffman et al., 2016). From a safety point of view, mRNA may also reduce the risk of mutagenesis related to pDNA; the chance of undesired insertion inserting into the genome is rare. Chemical structurally, however, single-stranded mRNA is less stable than a double-stranded plasmid. Moreover, mRNA may require a higher large-scale production cost than DNA (Youn and Chung, 2015).

Notably, siRNA could interrupt mRNA translation and reduce target gene expression by post-transcriptional silencing in homologous host genes (Fire et al., 1998). Fire and Melo, the Nobel Prize winners in 2006 (Fire et al., 1998), have elucidated such siRNA editing pathway. The siRNA mechanism of action has also been reported as a tool for preclinical study and disease therapy (Hannon, 2002). Briefly, short lengths of dsRNAs can be metabolized into siRNA by an endogenous ribonuclease (RNase) DICER in the intracellular molecule pathway. Then, miRNA is derived from the cleavage of dsRNA with a hairpin loop structure. In the cytoplasm, one strand of that single-stranded RNA (ssRNA) can then be bonded into a multiprotein complex known as the RNA-induced silencing complex (RISC). During the assembly process of RISC, one of the two strands of siRNA works as the guide strand to bind to the argonaute protein Ago2. Therefore, this guide strand guides RISC to incorporate its complementary target mRNA into the genome. The mRNA could then be cleaved by RNase (Pratt and MacRae, 2009), thus inhibiting the translation of the target gene.

Another kind of RNA, known as the short hairpin RNA (shRNA), is synthesized in the cell nucleus (as opposed to siRNA) and exported into the cytoplasm. The shRNAs can be recognized by the intracellular RNA interfering machinery and processed to be incorporated into RISC. A type of RNA that regulates the pools of genes of interest is called miRNA. The miRNA recruits RISC to form an RNA-RISC complex to incorporate mRNA that matches the sequence. The incomplete sequence match allows miRNA to bind to multiple mRNAs, leading to the degradation of an extensive range of potential effects (Iwakawa and Tomari, 2021). Compared to miRNA, siRNA binds to a single gene, thus demonstrating limited off-target effects and unwanted side effects (Stark et al., 1998).

In many cases, the elucidating of immunostimulatory properties is the final goal of gene delivery. Numerous nucleic acids could stimulate immune systems and elicit innate immune responses. Among them, the activation of immune defense mechanisms is found (Krysko et al., 2012). These nucleic acids, such as ssRNA in endosomes, CpG sequences, and mitochondrial DNA, are associated with ‘self’ damage-associated molecular patterns (DAMP)s in transmitting signals into immune cells (leukocytes, for example). This transmission is achieved through PRRs such as toll-like receptors (TLRs) (Chen et al., 2021a). These stimulations and cellular transmissions with cytokines and chemotaxis release would lead to overall cytokine release and proinflammatory protective effects. A balanced systemic anti-inflammatory activation response may be later created to restore tissue homeostasis. Minimizing off-target immune activation is necessary since even short siRNAs can mediate an innate immune response (Momen‐Heravi and Bala, 2018). Thus, engineering a nucleic acid sequence or delivery *via* effective vehicles could significantly facilitate on-target immune stimulations (Wolfrum et al., 2007).

ASOs are single-stranded DNAs or RNAs with 10–20 base pairs that could be complementary to target mRNA. ASOs modify protein translation and production by binding to their mRNA (Dias and Stein, 2002). The potential mechanism is that the bound ASOs could prevent translation factors from binding to the target mRNA and hinder the ribosome’s move to the mRNA strand. Another possible mechanism is that RNase H within the RNA-DNA duplex could cleave the RNA strand. Besides cytoplasm, ASOs also form triple helixes with DNA in the cell nucleus, leading to DNA cleavage and gene inhibition at the transcription state (Bajan and Hutvagner, 2020).

Various barriers exist to the delivery of nucleic acids. For instance, nucleic acids generally demonstrate a strong negative charge, high hydrophilic structure, and considerable molecule weight (10 kDa in siRNAs/miRNAs; 10^3^–10 (Jain et al., 2017) kDa in pDNA) (Durymanov and Reineke, 2018). Furthermore, immune recognition and clearance are challenging for the delivery of nucleic acids. These processes often take place in macrophages phagocytosis during blood circulation or face enzymes’ degradation, leading to unwanted immune response and production of cytokines. Even if nucleic acids have successfully avoided kidney excretion and extravasated out from the blood circulation, the final uptake by their target cell is a must. Due to the natural properties of nucleic acids, crossing the negatively-charged cellular membranes is challenging (Lee and Kataoka, 2011; Degors et al., 2019). Once inside the cell, nucleic acids face more barriers: to escape the endosome/lysosome; transport to the cytoplasm (for most RNAs) or cell nucleus (for pDNA), and incorporate into the target genes.

## 3 Nucleic acids as the cargo of NP delivery systems

Physically and chemically, various nucleic acids share similar properties and trafficking routes under extracellular and intracellular conditions. Therefore, many significant delivery barriers exist to them (Durymanov and Reineke, 2018; Mukalel et al., 2019). Herein, general challenges for nanocarriers-based nucleic acid delivery are further discussed.

### 3.1 Nucleic acid loading: Condensation and encapsulation

To protect nucleic acids from degradation or clearance from the environment and circulation, nanocarriers are generated from a broad range of materials (such as lipids and polymers) to facilitate their trafficking into ideal sites (Zhang et al., 2015a; Chen et al., 2016; Hu et al., 2016; Zou et al., 2016; Chen et al., 2017b; Hou et al., 2018; Chen et al., 2019). Many materials are naturally cationic charge; they could efficiently deliver the highly negatively-charged nucleic acids by electrostatic interaction. For instance, polymers with positively-charged amines could encapsulate or condense nucleic acids by electrostatic interactions; these polymers include polyethyleneimine (PEI), poly (l-lysine) (PLL), polyamidoamine (PAMAM), and poly (beta-amino ester)s (PBAEs), *etc* (Zhang et al., 2015b; Chen et al., 2015; Liu et al., 2018b; Luther et al., 2020; Barua and Pathak, 2022). This method also applies to cationic liposomes, whose spherical structure contains both hydrophilic and hydrophobic moieties with positively-charged lipids. The design of cationic liposomes would efficiently facilitate their binding to the hydrophilic nucleic acids (Ball et al., 2018a).

On the other hand, nucleic acids could chemically conjugate to nanocarriers by bioconjugation thanks to several of their active bioconjugation sites. Bioconjugation reactions (Chen et al., 2020a), such as amine conjugation, thiol-based conjugation, and azide-alkyne cycloaddition reactions (Ruan et al., 2018), could help nucleic acids attach to the surface of NPs. Notably, large nucleic acids such as pDNA and mRNA have a higher density of negative charge that may enhance the stability of lipid and polymer nanocarriers when conjugating. In turn, nucleic acids can hardly be released from the carriers due to these strong interactions (Liu, 2019; Gómez-Aguado et al., 2020). Therefore, choosing the proper formulation may help balance the delivery system’s stability and nucleic acids’ release. For instance, shortening the polymer chain’s length, reducing the hydrophobicity, and enhancing the charge density of the materials could highly increase the chance of nucleic acid release. However, it could also likely decrease the complex stability. Otherwise, the nucleic acids are hardly released, but the stability of the delivery complex is compromised.

### 3.2 Cellular internalization of nucleic acids

The endocytic pathways of nanocarrier-genetic cargo complexes are critical for nucleic acids to employ their gene transfection functions. Encapsulated nucleic acids typically cross the cellular membrane by endocytosis. Based on the type of cytoplasmic proteins involved, there are several different endocytic pathways, including clathrin- and caveolae-dependent endocytosis, clathrin-/caveolin-independent endocytosis, and micropinocytosis (van den Berg et al., 2021). In addition, nucleic acids rely on the properties of the nanocarriers-nucleic acid complexes for endocytosis, such as NPs’ formulation material, size, surface charge, and shape (Rennick et al., 2021).

### 3.3 Endosomal escape

Upon internalization, NPs enter into the endosome/lysosome. Nucleic acids as immunostimulatory agonists would stimulate receptors on the endosome. For instance, dsRNA could bind to TLR3, ssRNA could bind to TLR7/8, and the unmethylated CpG sequences could bind to TLR9 (Huang et al., 2021; Zou et al., 2022). However, most nucleic acids such as pDNA, mRNA, and RNA oligos are not purposed to trap in endosomes. These nucleic acids must be degraded in the endosomes and escaped/trafficked into the cytoplasm or cell nuclei to be functionally effective.

Since endosomes and cellular plasma membranes share similar lipid components, the NP-endosome interaction during endosomal escape employs the exact mechanism of cellular internalization (Ma, 2014). However, the endosome has an acidic environment compared with the neutral environment of the cell. Therefore, without NP protection, most nucleic acids end up in endosome/lysosomes during enzymatic digestion after endocytosis (Cupic et al., 2019). Thus, the nucleic acid endosomal escape is essential in improving genetic therapeutics’ efficiency. Furthermore, depending on the NPs’ properties, different endosomal escape mechanisms are involved in trafficking nucleic acids entering the cytoplasm, such as pH-triggered membrane disruption (Rietwyk and Peer, 2017) and umbrella effect (Behr, 1997), fusion with the endosomal membrane, and pore formation (Ahmad et al., 2019).

### 3.4 Nucleic acid release from the nanocarriers

The physical and chemical properties of different nanocarrier materials determine the release capability of nucleic acids. Upon NPs’ endosomal escape, nucleic acids (such as mRNA and siRNA) need to be released from the nanocarriers to employ functions in the cytoplasm and act on protein translation. However, some nucleic acids, such as pDNA, should be further carried and protected until delivered to the cell nucleus (Nguyen and Szoka, 2012; Chen et al., 2020b). Lipid-based nanocarriers in lipoplexes can interact with the endosome membrane, causing membrane fusion or disruption.

On the other hand, the interaction may facilitate their endosomal escape, and nucleic acids could be released from lipid-based nanocarriers (Bauhuber et al., 2009). In turn, lipoplexes would be suitable for the delivery of nucleic acids that act in the cytoplasm. Nucleic acids are generally released from the polymer-based nanocarriers in polyplexes by anionic exchange with genomic DNA. The balance of the nucleic acid release and the stability of polymer-based nanocarriers is critical, which might be controlled by various properties of materials. For instance, reducing the chain length, decreasing the hydrophobicity, or enhancing the charge density of polymer would reduce nanocarriers’ stability but increase the chances of nucleic acid release. Otherwise, the nucleic acids can hardly be released, and the stability is compromised (Kanvinde et al., 2022).

## 4 Barriers to oral nucleic acid delivery

Local gene delivery is the application of delivering nucleic acid-encapsulated nanocarriers directly into the target tissue without getting into the circulation, thus increasing cargo bioavailability and reducing side effects by minimizing unwanted distribution to other tissue sites. In addition, condensed distribution of the local delivery requires fewer administrations but sufficient treatment effectiveness (Kriegel et al., 2013).

In this scenario, oral administration is a highly attractive route of administration from both the manufacturing and patients’ perspectives. Oral delivery offers many advantages, including non-sterile manufacturing requirements, high patient compliance, and avoidance of needle-associated discomfort. However, the GI tract presents a harsh environment with various biological barriers to macromolecule absorption. At this point, nano drug delivery platforms are developed to enable adequate target access.

The primary target of oral delivery is the GI tract. GI tract represents a large surface area with facilitated nucleic acid uptake thanks to its numerous microvessels. Therapeutic cargo is further delivered into diseased cells within the bloodstream. Oral delivery could be promising for treating GI diseases such as IBD because of the direct contact of ingested materials with diseased cells (Attarwala et al., 2018). Another promising approach to oral delivery is pDNA-based vaccines targeting M-cells. M-cells in the GI tract act as the entry portal for pathogens. Thus targeting M-cells facilitates therapeutic cargo crossing the mucosal and systemic immunological barriers in entering the targets (O’Driscoll et al., 2019). In 1999, a DNA-based NP system was developed, whereas DNA encoding for the peanut allergen gene has been shown to transduce gene translation in the epithelium of mucosal and induce systemic immunity upon oral administration of this DNA vaccine (Li et al., 1999). However, the challenge of oral delivery is that various degrading enzymes in the intestine may lead to insufficient antigen uptake and low transfection efficiency (El‐Mayta et al., 2021).

Compared to other routes of administration, the advantage of oral nucleic acid administration is its non-invasiveness, ease of administration, good patient compliance, and convenience. Moreover, compared to systemic nucleic acid administration, targeted/localized nucleic acid oral delivery into the GI tract could increase bioavailability and reduce side effects (Durán‐Lobato et al., 2020). However, most oral non-viral delivery of nucleic acids has shown a relatively low efficiency of transfection (Bourgeois et al., 2005).

Since the oral administration route is not as straightforward, the nucleic acid payload must face several physical barriers in reaching desired sites and cells. Many barriers exist, such as numerous proteolytic enzymes at the brush border membrane and in the gut lumen, the mucus layer, the bacterial gut flora, and the epithelium (Yamanaka and Leong, 2008). For example, low pH in the gut lumen helps enzymes degrade nucleic acids, leading to low efficiency in nucleic acids reaching the targeted cells. In addition, unlike systemic delivery, the mucous and epithelial cell layers are massive physical barriers preventing nucleic acids from evading phagocytosis and being taken up by the targeted cell (Durán‐Lobato et al., 2020).

### 4.1 Oral deliveries of therapeutics in inflammatory bowel disease

**FIGURE 2:**
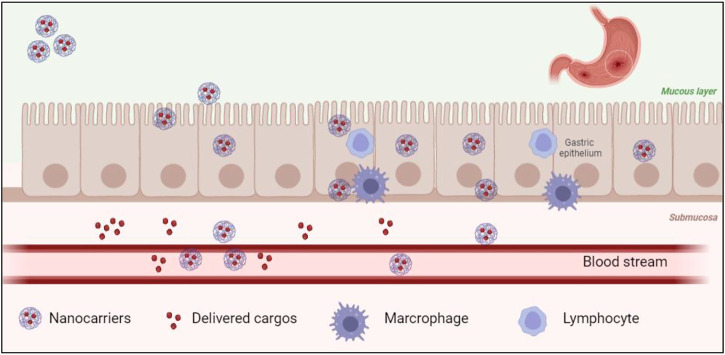
Biological barriers exist in nano-cargo delivery at the GI tract.

IBD consists of Crohn’s disease and UC and is commonly caused by mucosal infection and inflammation in the GI tract (Bouma and Strober, 2003; Strober et al., 2007). To improve the safety and efficacy of current treatment approaches, nucleic acid treatment has been largely investigated (Chen et al., 2021b). Tumor necrosis factor (TNF)-α treatment is one of the methods that has been applied in treating IBD. TNF-α is a cytokine known as the inflammatory mediator. However, direct cytokine treatment may cause severe side effects such as infusion reactions, opportunistic infections, and antibody formation, eventually decreasing the efficiency of such cytokine treatment (Papa et al., 2009). Therefore, gene therapies have recently been utilized as a novel treatment option for many GI tract diseases such as IBD and viral infection. However, oral delivery of TNF-α to the GI tract (Figure 2) is still challenging. Preventing nucleic acid degradation by enzymes from the gastric and intestinal epithelium is essential; increasing nucleic acid transpassing the submucosa into the bloodstream and uptake into diseased cells is also a must.

So far, NP-in-microsphere oral system for gene delivery has shown an enhanced vehicle residence time, increased vehicle accumulation, and nucleic acid release in the large intestine (Whitehead et al., 2009). In a dextran sodium sulfate (DSS)-induced UC-bearing BALB/c murine IBD model, oral TNF-α siRNA was administrated. In this NP-in-microsphere delivery system, TNF-α siRNA was successfully encapsulated (Kriegel and Amiji, 2011a).

An ASO called Mongersen that inhibits the TNF-α pathway has been shown in phase II clinical trials to relieve remission in patients with active Crohn’s disease. However, because of the apparent inefficacy of the treatment, the phase III clinical trial failed. Therefore, many new therapeutic approaches with nanocarriers have been recently studied to target TNF-α. In a DSS-induced inflammatory murine model, an ASO-encapsulated chitosan hydrogel (83.5% loading efficiency) was orally-administered (Duan et al., 2019). Results indicated that ASO was successfully released to the inflamed intestine and taken locally by intestinal cells and macrophages. In addition, the cell-specific targeting demonstrated a significant local downregulation of TNF-α, myeloperoxidase, and malondialdehyde (Duan et al., 2019).

Another approach to reducing the TNF-α signal is centered on siRNAs complexed with cationic polyplexes or lipoplexes. For example, a siRNA targeting the Map4k4 gene involved in the TNF-α pathway was encapsulated into an oral delivery system (PEI with shells). Results demonstrated a significantly improved mice survival rate and 80% inhibition of TNF-α production after lipopolysaccharide (LPS)-induced intraperitoneal injection. In another study, an anti-TNF-α siRNA was loaded into thioketal NPs to trigger-release the nucleic acids in response to reactive oxygen species (ROS) (Aouadi et al., 2009). After oral administration of these NPs to the UC-induced murine IBD model, a significantly-reduced mRNA level of TNF-α was found in the colon, and levels of IBD inflammation were also diminished (Wilson et al., 2010).

In treating IBD, mannosylated-NPs have also been studied to target macrophages for the oral delivery of nucleic acids. A study has successfully coloaded anti-TNF-α siRNA and pro-healing interleukin (IL)-22 into mannosylated-NPs for macrophage targeting. These NPs were mixed with chitosan hydrogel to create an oral delivery system in treating DSS-induced colitis mice (Xiao et al., 2013). As a result, key colitis markers (colon length, myeloperoxidase level, histological score, and TNF-α protein expression) were recovered to the levels of healthy controls (Xiao et al., 2018).

Researchers have evaluated lipid NPs’ fate and functions in the GI tract and found that lipid NPs could protect and promote siRNA delivery into epithelial cells in the intestinal (Ball et al., 2018b). In addition, to selectively silencing CyD1 in leukocytes *in vivo*, hyaluronan-labeled lipid vesicles were loaded with CyD1 siRNA and successfully delivered at mouse colitis sites. Results demonstrated a significant reduction of gut inflammation and TNF-α and IL-12 expressions (Peer et al., 2008). In another study, CD98 siRNA-loaded ginger-derived nano-lipids were orally administrated in treating UC. These siRNAs were effectively and selectively targeted to colon tissue, significantly reducing CD98 expression on site (Zhang et al., 2017).

In a DSS-induced acute colitis murine model, a novel oral delivery system was developed based on poly (caprolactone) microspheres with siRNA-loaded gelatin. This multi-compartmental platform combinational delivered siRNAs in targeting both TNF-α and cyclin D1. As a result, significant reductions in gene expression of TNF-α and CyD1 in colon cells and other proinflammatory cytokines were found (Kriegel and Amiji, 2011b). In addition, the murine model showed significantly-recovered body weight and a reduction in colon myeloperoxidase activities. A summary of more comparable findings is listed below in Table 2.

**TABLE 2:** Animal models and clinical trials of nucleic acid-based gene therapeutics for IBD.

Delivery system	Administration route	Cargo	Animal model	Citation
polysaccharide-encapsulated colon-specific hydrogel nanocomposites	oral	TNF-α/phosphorothioated-ASOs	DSS-induced inflammation in mice	Duan et al. (2019)
PEI polyplexes in β1,3-D-glucan shells	oral	Map4k4/siRNA	LPS-induced inflammation in mice	Aouadi et al. (2009)
poly (lactic acid)(PLA)-loaded with CD98 siRNA	oral	CD98/siRNA	DSS-induced inflammation in mice	Laroui et al. (2014)
CD98-poly (ethylene glycol)(PEG)-urocanic acid-modified chitosan NPs carrying CD98 siRNA	oral	CD98/siRNA	DSS-induced inflammation in mice	Xiao et al. (2014)
ROS-sensitive thioketal NPs	oral	TNF-α/siRNA	DSS-induced inflammation in mice	Wilson et al. (2010)
HA-functionalized CD98 siRNA/curcumin-loaded (poly (lactide-co-glycolide)(PLGA) NPs encapsulated in a chitosan/alginate hydrogel	oral	CD98/siRNA +curcumin	DSS-induced inflammation in mice	Xiao et al. (2016)
galactose-functionalized TNF-α siRNA-loaded PLGA NPs embedded in a chitosan/alginate hydrogel	oral	TNF-α/siRNA +IL-22	DSS-induced inflammation in mice	Xiao et al. (2018)
gelatin NPs encapsulating a combination of siRNA duplexes	oral	TNF-α/siRNA CyD1/siRNA	DSS-induced inflammation in mice	Kriegel and Amiji, (2011b)
galactosylated trimethyl chitosan–cysteine NPs	oral	Map4k4/siRNA	DSS-induced inflammation in mice	Zhang et al. (2013)
PEI-polylactide NPs	oral	TNF-α/siRNA	DSS-induced inflammation in mice	Laroui et al. (2011)
hydrogel composed of alginate and chitosan	oral	krüppel-like factor 4 (KLF4)/siRNA	DSS-induced inflammation in mice	Ghaleb et al. (2014)
Mongersen	oral	suppressor of mothers against decapentaplegic homolog 7(SMAD7)/ASO	Crohn’s disease in Phase III trial	Sands et al. (2020)

### 4.2 Oral nuclei acid therapy against respiratory viral infections

In March 2020, the world faced a severe pandemic related to COVID-19 (Lu et al., 2020) caused by infection of severe acute respiratory syndrome coronavirus 2 (SARS CoV-2). Over 79 million people and 1.7 million deaths were reported by the World Health Organization (WHO) (Chen et al., 2020c). So far, the only treatment authorized by the Food and Drug Administration (FDA) in targeting SARS-CoV-2 is the nucleoside analog GS-5734 (remdesivir), a non-obligate RNA chain terminator. Irrespective of disease severity, remdesivir demonstrated significantly reduced hospital recovery time for adults and children (Cowling et al., 2020; Gibney, 2020; Lee and Choi, 2020). Besides human data, the preclinical studies also confirmed a lower lung vial production and lung pathology 12h after GS-5734 was administered in SARS-CoV-2 infected animals. However, GS-5734 remains challenging in controlling high-risk exposure since it can only be administered by the intravenous route (Beigel et al., 2020; US Food and Drug Administration, 2020). Therefore, oral delivery of nucleic acids might become a promising strategy, although only limited research has been conducted so far.

For instance, MK-4482 (molnupiravir) is a broad activity inhibitor of influenza A and respiratory syncytial viruses. It acts as a bioavailable prodrug (5-isobutyric ester form) of cytidine nucleoside analog EIDD-1931 (β-D-N (Vaidya et al., 2019)-hydroxycytidine) and functions primarily as RNA-based mutagenesis when orally-administrated (Toots et al., 2019). In the early 2000s, MK-4482 was initially developed for hepatitis C virus treatment. A recent study has shown that EIDD-1931 demonstrated potent activity against MERS-CoV-1, SARS-CoV-1, and SARS-CoV-2 coronaviruses in multiple human cell lines and mouse models (Kwon et al., 2021; Rosenke et al., 2021). Moreover, another study on the highly-susceptible Syrian hamster model depicted that when the model was oral administration with MK-4482 and then SARS-CoV-2 infected in a 12h timeline, the model showed significantly decreased viral lung loads and lung pathology, although kept shedding from the upper respiratory tract (Yoon et al., 2018). MK-4482 has also demonstrated its potential as an oral administration drug for high-risk infections. Ridgeback and Merck developed MK-4482 with an active antiviral ingredient β-d-N4-deoxycytidine for oral use. The β-d-N4-deoxycytidine could induce lethal mutation during RNA synthesis by pairing with adenine and guanine (Malone and Campbell, 2021). After MK-4482 treatment, hospitalization and mortality of COVID-19 patients significantly decreased by nearly 50% (Malone and Campbell, 2021).

Compared to remdesivir, MK-4482 showed a more potent antiviral effect and lower toxicity among *in vitro* tests (Sheahan et al., 2020). The phase II clinical trial result of MK-4482 treatment showed that after five days of 400 mg or 800 mg treatment, no live virus was identified among treated patients. Safety and tolerability profiles were also significantly improved. MK-4482 is currently in phase II/III clinical trials based on encouraging preclinical data and minor toxicity and adverse side effects in phase I clinical trials. However, MK-4482 could become a paradigmatic example of using lethal mutagenesis as an antiviral strategy. It is a mutagenic ribonucleoside and can be carcinogenic (Fischer et al., 2021). Inherent risks exist for MK-4482, as EIDD-1931 can be metabolized by the host cell to 2′-deoxyribonucleoside form by ribonucleotide reductase and then incorporated into the host cell’s DNA. The mutagenic effect of EIDD-1931 has been shown in animal cell cultures, and overexposure to MK-4482 may cause long-term ill effects such as cancer and potentially lead to congenital disabilities in embryo development or detrimental mutations in sperm precursor cells. Further studies in practical and economic oral anti-COVID-19 drugs and vaccines are still in progress and are heavily conducted, as no solid studies are settled yet (Fan et al., 2022).

A branched hybrid poly (β-amino ester) mRNA NP flatform was developed and capsule-mediated orally dosed to GI tissue, enabling nucleic acid delivery to stomachs for both gastric and systemic uptake and transfection. Similar to vaccines, this platform would facilitate rapid utilization of episodic interventions (Abramson et al., 2022). Moreover, a self-amplifying RNA lipid NP flatform was deployed to neutralize severe acute SARS-COV-2 (variants alpha and delta). After treating the murine model with this oral vaccine, S-protein was successfully expressed at both mRNA and protein levels. Higher titers of IgG and IgA were also observed in week 6. The wild-type viral neutralization assay demonstrated that vaccinated mice (with recovered COVID-19 patients’ secreted antibodies) could neutralize SARS-COV-2 variants alpha and delta (Keikha et al., 2021; Schooley et al., 2021). Future validations for these studies are also in urgent need.

Since the pandemic, effective drugs treating and preventing COVID-19 have been in high demand. Although effective, Remdesivir has shown limitations in clinical intravenous administration; their concentrations in plasma vary; the antiviral activity is also unstabble (Schooley et al., 2021). The FDA has approved four neutralizing antibodies for treating COVID-19: casirivimab, bamlanivimab, etesevimab, and imdevimab. However, due to the high cost and requirements for intravenous administration of these antibodies, they are limited for broader public use. Therefore, affordable and powerful oral drugs for the prevention and treatment of COVID-19 might be a possible way of solution.

## 5 Challenges in translational oral delivery of nucleic acids

To succeed in the transition from lab bench work to clinical development, many challenges of oral nucleic acid formulation must be overcome. Controlled/triggered vehicle design, robustness, and safety concerns are critical (Hua et al., 2015). In current clinical trials, the standard guideline for formulation development is to seek a simplified drug delivery design. Moreover, the excipients in the formulation should be FDA-approved or regarded as generally recognized as safe (GRAS) (Morales et al., 2017). Lipid-based nanocarriers are full of potential based on their well-known materials for formulation designs and their effectiveness in nucleic acid delivery. However, this emerging strategy increases concerns about toxicity, regulation, cost, scale-up feasibility, and controlled performance (Bobo et al., 2016). Strict quality control and translational research design are in urgent need. In this case, the nanotechnology research field has published a guideline for the clinic: “minimum information standardization” for bio-nano systems (Faria et al., 2018).

## 6 Conclusion

The sustainable development of oral nucleic acids’ delivery for the treatment of infections relies heavily on new molecular biology, chemistry, and engineering studies. Notably, gene targets and nanocarriers’ emerging findings offer great opportunities for significant discoveries. In addition, new promising drug delivery systems could overcome multiple barriers to progressing oral nucleic acid therapeutics in the clinic. In treating infectious diseases, lessons continue to be learned from translational nanomaterial and nucleic acid research.

## Glossary

ASO: antisense oligonucleotide
COVID-19: coronavirus disease 2019
DAMP: damage-associated molecular pattern
DNA: deoxyribonucleic acid
dsRNA: double-stranded ribonucleic acid (RNA)
DSS: dextran sodium sulfate
FDA: Food and Drug Administration
GI tract: gastrointestinal tract
gRNA: guide RNA
GRAS: generally recognized as safe
HA: hyaluronic acid
IBD: inflammatory bowel disease
IL-22: interleukin-22
KLF4: krüppel-like factor 4
LPS: lipopolysaccharide
mRNA: messenger RNA
miRNA: microRNA
NP: nanoparticle
PAMAM: polyamidoamine
PBAE: poly(beta-amino ester)
pDNA: plasmid DNA
PEG: poly(ethylene glycol)
PEI: polyethyleneimine
PLA: poly(lactic acid)
PLGA: poly(lactide-co-glycolide)
PLL: poly(l-lysine)
PRR: pattern-recognition receptor
RISC: RNA-induced silencing complex
RNA: ribonucleic acid
RNase: ribonuclease
ROS: reactive oxygen species
SARS CoV-2: severe acute respiratory syndrome coronavirus 2
shRNA: short hairpin RNA
siRNA: small interfering RNA
SMAD7: suppressor of mothers against decapentaplegic homolog 7
ssRNA: single-stranded RNA
TLR: toll-like receptor
TNF: tumor necrosis factor
UC: ulcerative colitis
WHO: World Health Organization
